# Progress in Allogeneic Hematopoietic Cell Transplantation in Adult T-Cell Leukemia-Lymphoma

**DOI:** 10.3389/fmicb.2019.02235

**Published:** 2019-10-01

**Authors:** Atae Utsunomiya

**Affiliations:** Department of Hematology, Imamura General Hospital, Kagoshima, Japan

**Keywords:** ATL, HTLV-1, allogeneic hematopoietic cell transplantation, graft-versus-ATL effect, HTLV-1 proviral load, molecular targeting agent, mogamulizumab, immunotherapy

## Abstract

The prognosis of aggressive adult T-cell leukemia-lymphoma (ATL) remains poor because of frequent infections and drug resistance. Dose-intensified chemotherapy followed by autologous stem cell transplantation failed to improve the prognosis of patients with ATL; however, we first revealed that allogeneic hematopoietic cell transplantation (allo-HCT) might improve their prognosis. We showed that reduced-intensity stem cell transplantation using peripheral blood was feasible for elderly patients. Further, the prognosis of patients in remission, who receive cord blood transplantation, has been recently improved and is equivalent to that of patients who receive transplants from other stem cell sources. As for the timing of HCT, the patients who underwent transplantation early showed better outcomes than those who underwent transplantation late. Based on the analysis of patients with aggressive ATL, including those who received transplants, we identified five prognostic factors for poor outcomes: acute-type ATL, poor performance status, high soluble interleukin-2 receptor levels, hypercalcemia, and high C-reactive protein level. Next, we developed a new prognostic index: the modified ATL-PI. The overall survival (OS) rates were significantly higher in patients who underwent allo-HCT than those who did not in the intermediate and high-risk groups stratified using the modified ATL-PI. Two new anti-cancer agents, mogamulizumab and lenalidomide, were recently approved for ATL patients in Japan. They are expected to induce longer survival in ATL patients when administered along with transplantation. However, a retrospective analysis that the risk of severe, acute, and corticosteroid-refractory graft-versus-host disease was higher in patients who received mogamulizumab before allo-HCT, and that mogamulizumab might increase the transplant-related mortality (TRM) rates and decrease the OS rates compared to those of patients who did not receive mogamulizumab. However, our recent study showed that administration of mogamulizumab before allo-HCT tended to improve the survival of patients with ATL. In conclusion, allo-HCT procedures for patients with aggressive ATL have considerably progressed and have helped improve the prognosis of these patients; however, many concerns still remain to be resolved. Further development of allo-HCT by using new molecular targeting agents is required for the improvement of cure rates in patients with ATL.

## Introduction

Adult T-cell leukemia-lymphoma (ATL) is an intractable peripheral T-cell malignancy caused by human T-cell leukemia virus type-I (HTLV-1). The overall survival (OS) rates by conventional chemotherapy are very low because of drug resistance for anti-cancer agents and susceptibility for various kinds of infectious diseases (Ishitsuka and Tamura, 2014; Utsunomiya et al., 2015).

Dose-intensified chemotherapy followed by autologous stem cell transplantation failed to improve the prognosis of patients with ATL because of high ATL relapse rates and frequent infectious complications (Asou et al., 1985; Tsukasaki et al., 1999). Conversely, the successful treatment of a patient with aggressive ATL with allogeneic hematopoietic cell transplantation (allo-HCT) was reported in 1987. However, the patient died from interstitial pneumonitis due to cytomegalovirus (CMV) infection 117 days after transplantation (Sobue et al., 1987). Subsequently, several ATL cases that were successfully treated using allo-HCT were reported (Ljungman et al., 1994; Borg et al., 1996; Obama et al., 1999; Kishi et al., 2001). In 2001, we first reported the possibility of improved outcomes in patients with aggressive ATL by using allo-HCT (Utsunomiya et al., 2001). Although the prognosis of patients with aggressive ATL improved by allo-HCT, transplant-related mortality (TRM) rates in patients who received transplantation with myeloablative conditioning (MAC) regimen were very high (Utsunomiya et al., 2001; Kami et al., 2003; Fukushima et al., 2005). However, the TRM rates of patients who received reduced-intensity stem cell transplantation (RIST) decreased compared to those of patients transplanted using MAC (Tanosaki et al., 2008). Although the prognosis of patients with ATL who underwent cord blood transplantation (CBT) was poorer than that of patients transplanted with other stem cell sources, it has recently improved (Hishizawa et al., 2010; Nakamura et al., 2012; Fukushima et al., 2013; Kato et al., 2014).

The Japan Society for Hematopoietic Cell Transplantation established a hematopoietic cell transplantation registry program named Transplant Registry Unified Management Program (TRUMP; Atsuta et al., 2007). According to this Japanese TRUMP database, approximately 2000 patients with ATL who had undergone allo-HCT were enrolled until 2017 in Japan (The Japanese Data Center for Hematopoietic Cell Transplantation/The Japan Society for Hematopoietic Cell Transplantation, 2017)^1^. Conversely, in countries other Japan, such as Europe, only a small number of patients with ATL underwent allo-HCT [retrospective study by Bazarbachi et al. (2014)].

At present, allo-HCT is thought to be the only potential curative treatment for patients with aggressive ATL. In this study, I review the progresses in allo-HCT for ATL in Japan.

## Development of Allo-HCT for ATL

### Stem Cell Sources

Bone marrow cells, peripheral blood stem cells, and cord blood cells are generally used as stem cell sources for allo-HCT for patients with ATL like in other hematological malignancies. A nationwide survey in Japan revealed that the OS rates of patients with ATL receiving CBT were lower than those of patients receiving bone marrow transplantation (BMT) or peripheral blood stem cell transplantation (Hishizawa et al., 2010; Ishida et al., 2012a). Subsequently, two retrospective studies showed that the prognosis of patients with ATL in remission who underwent CBT had improved and was equivalent to that of patients who were transplanted with other stem cell sources, such as bone marrow cells or peripheral blood stem cells (Table 1; Nakamura et al., 2012; Fukushima et al., 2013). Recently, in Japan, the number of patients with ATL receiving CBT has rapidly increased because available related donors have markedly decreased, and coordination time for allo-HCT in unrelated transplantation is too long, which hinders early transplantation at an appropriate time. The analysis of the Japanese TRUMP database revealed that prognosis of patients with ATL who underwent CBT has gradually improved recently. The TRM rates of patients with ATL in remission who underwent CBT decreased. By decreasing the TRM rates, the OS rates of patients with ATL who underwent CBT improved like those of patients transplanted with other stem cell sources (Table 1). If patients with ATL receiving allo-HCT have suitable related donors, a stem cell source, BM, or PB, would be chosen considering patient age, disease status, and performance status. Early transplantation from alternative donors, such as cord blood or HLA haplo-identical donor would be recommended if a patient does not have suitable related donors for allo-HCT. As the Japan Cord Blood Bank Network supplies cord blood units to recipients undergoing CBT, cord blood is the most appropriate stem cell source in Japan.

**TABLE 1:** Cord blood transplantation for adult T-cell leukemia-lymphoma.

**References**	**Pt. No.**	**Median age (range)**	** Sex M/F**	**Subtype**	**Disease status at CBT**	**Conditioning regimen**	**Cause of death**	**Outcome**
Tamura et al., 2006	11	56 (39–61)	Unk	Unk	CR: 2 Non-CR: 9	MAC: 2 RIC: 9	TRM: 5 ATL: 5	Unk
Hishizawa et al., 2010	90	Unk	52/38	Unk	CR: 26 Non-CR: 57 Unk: 7	MAC: 14 RIC: 64 Unk: 12	TRM: 45 ATL: 25 Unk: 4	3Y-OS: 17% (95% CI 9–25)
Nakamura et al., 2012	10	51 (31–64)	6/4	Acute: 9 Lymphoma: 1	CR: 2 PR: 4 SD: 1 PD: 3	MAC: 6 RIC: 4	ATL: 4 Sepsis: 1 GVHD + ATL: 1	2Y-OS: 40% (95% CI 67–12)
Ishida et al., 2013	174	Unk	Unk	Unk	Unk	Unk	Unk	3Y-OS: 21% (95% CI 15–29)
Fukushima et al., 2013	27	52 (41–63)	18/9	Acute: 17 Lymphoma: 10	CR: 5 PR: 11 PIF: 5 REL: 6	MAC: 9 RIC: 18	TRM: 10 ATL: 9	3Y-OS: 27.4%
Kato et al., 2014	175	55 (27–79)	105/70	Unk: 175	CR: 50 Non-CR: 116 Unk: 9	MAC: 63 RIC: 108 Unk: 4	TRM: 77 ATL: 52	2Y-OS: 20.6% (95% CI 14–27)
Choi et al., 2016	15	62 (55–69)	8/7	Acute: 13 Lymphoma: 2	CR: 5 PR: 10	RIC: 15	TRM: 3 ATL: 4	2Y-OS: 53.3%
Tokunaga et al., 2018	150	61 (24–78)	88/62	Acute: 107 Lymphoma: 39 Other: 4	CR: 62 PR: 86 Unk: 2	MAC: 54 RIC: 95 Unk: 1	TRM: 54 ATL 46	1Y-OS: 38.3% (95% CI 30–47)

*ATL, adult T-cell leukemia-lymphoma; CBT, cord blood transplantation; CR, complete remission; PR, partial remission; SD, stable disease; PD, progressive disease; PIF, primary induction failure; REL, refractory after relapse; OS, overall survival; MAC, myeloablative conditioning; RIC, reduced intensity conditioning; TRM, transplant-related mortality; GVHD, graft-versus-host disease; Unk, unknown.*

### Indication of Allo-HCT for ATL

**FIGURE 1:**
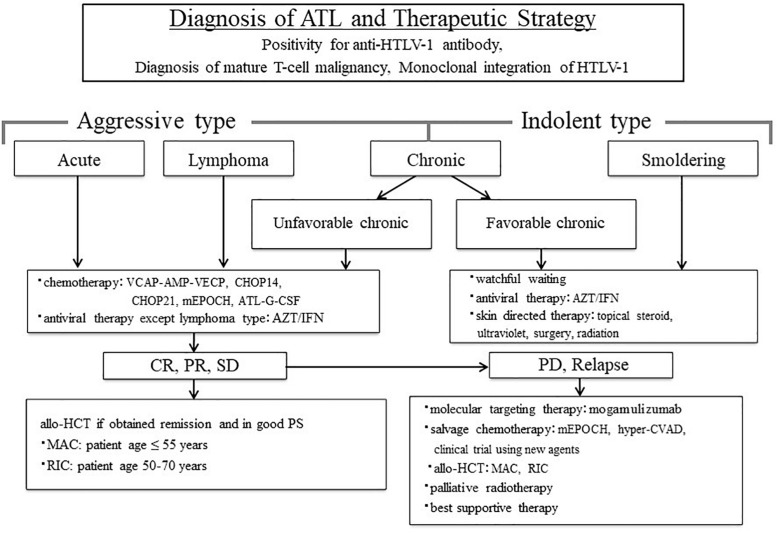
Treatment algorithm for adult T-cell leukemia-lymphoma (ATL) patients. The figure is reproduced modified from Figure 1 in Utsunomiya et al. (2015). ATL diagnosis is based on anti-HTLV-1 antibody positivity in the serum, the presence of mature T-cell malignancy, and the Southern blot detection of monoclonal integration of HTLV-1 proviral DNA in the tumor cells. ATL treatment is usually determined according to the clinical subtypes and prognostic factors. The presence of an aggressive-type ATL (acute, lymphoma, and unfavorable chronic types) or indolent-type ATL (favorable chronic and smoldering types) is critical to make treatment decisions. Patients with an aggressive-type generally receive immediate combination chemotherapy or antiviral therapy with zidovudine and interferon-α (AZT/IFN), except for those with lymphoma-type ATL. The international consensus meeting primarily recommends the VCAP–AMP–VECP regimen. Other therapeutic regimens include CHOP14, CHOP21, mEPOCH, and ATL-G-CSF. Patients undergo further treatment with allogeneic hematopoietic cell transplantation, which is particularly effective in young patients with good performance status, and those who have achieved remission before transplantation. In Japan, patients with an indolent-type ATL without any skin lesions are usually followed-up under a watchful waiting policy until the disease transforms to an aggressive type. Antiviral therapy is frequently used for patients with favorable chronic and smoldering type of ATL in non-Japanese nations, and skin-directed therapy is applied for smoldering ATL with skin manifestations. allo-HCT, allogeneic hematopoietic cell transplantation; ATL-G-CSF, combination chemotherapy consisting of vincristine, vindesine, doxorubicin, mitoxantrone, cyclophosphamide, etoposide, ranimustine, and prednisone with granulocyte-colony stimulating factor support; AZT/IFN, zidovudine and interferon-α; CHOP, cyclophosphamide, doxorubicin, vincristine and prednisone (CHOP14 is performed every 2 weeks, and CHOP21 is performed every 3 weeks); CR, complete remission; hyper-CVAD, cyclophosphamide, vincristine, doxorubicin, and dexamethasone; MAC, myeloablative conditioning; mEPOCH, etoposide, prednisone, vincristine, cyclophosphamide, and doxorubicin (EPOCH) with modifications; PD, progressive disease; PR, partial remission; PS, performance status; RIC, reduced-intensity conditioning; SD, stable disease; VCAP–AMP–VECP, vincristine, cyclophosphamide, doxorubicin and prednisone (VCAP)–doxorubicin, ranimustine and prednisone (AMP)-vindesine, etoposide, carboplatin, and prednisone (VECP). Reprinted with permission of Cancer Science.

Before treatment, the clinical subtypes of ATL should be determined after ATL diagnosis (Shimoyama, 1991). The therapeutic strategy for patients with ATL can be decided by further dividing ATL into two categories: aggressive type, including acute, lymphoma and unfavorable chronic type; and indolent type, including favorable chronic and smoldering type (Shimoyama, 1994; Tsukasaki et al., 2009). We proposed the treatment algorithm for patients with ATL based on these clinical subtypes and clinical characteristics (Figure 1; Utsunomiya et al., 2015). All young patients with ATL who obtained complete remission (CR), partial remission (PR), and stable disease (SD) by chemotherapy and maintained good performance status (PS) were recommended for allo-HCT. Even in relapsed patients with ATL who underwent chemotherapy, the OS rate in patients who received allo-HCT after relapse was higher than that in patients who did not (Fuji et al., 2018b). This suggests that allo-HCT is one of the promising salvage therapies for even relapsed patients with ATL.

**FIGURE 2:**
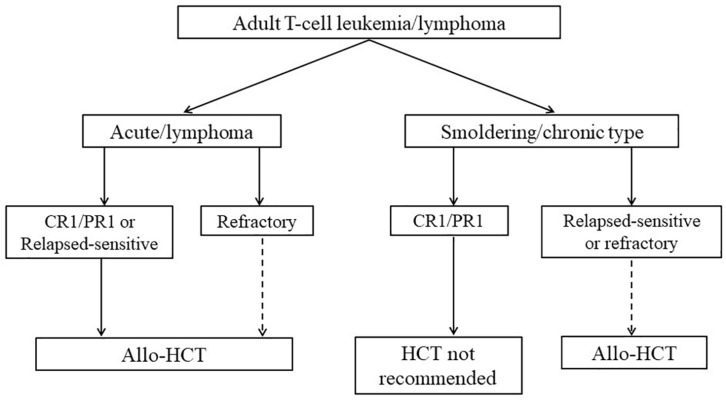
Algorithm summarizing indications for HCT in ATL. The figure is reproduced from Figure 5 in Kharfan-Dabaja et al. (2017). The dashed line denotes a weak recommendation. CR, complete remission; PR, partial remission; allo-HCT, allogeneic hematopoietic cell transplantation. Reprinted with permission of Biology of Blood and Marrow Transplantation.

Recently, the Guidelines Committee of the American Society for Blood and Marrow Transplantation proposed clinical practice recommendations in mature T cell and NK-T cell lymphomas, which include indication and timing of HCT for patients with ATL (Figure 2; Kharfan-Dabaja et al., 2017). These recommendations of transplantation for patients with ATL are similar to our proposal of indications for allo-HCT (Utsunomiya et al., 2015; Kharfan-Dabaja et al., 2017). Furthermore, the most recent International Consensus Meeting Report strongly recommends up-front allo-HCT for all suitable patients with aggressive ATL (Cook et al., 2019).

Concerning donor eligibility criteria, the use of HTLV-1 carriers as donors in allo-HCT for ATL patients is controversial. Although HTLV-1 carriers are omitted for donors in unrelated transplantation and CBT, they are not excluded for donors in related transplantation. When allo-HCT is planned for patients with ATL by using an HTLV-1 carrier donor, no monoclonal or oligoclonal integration by Southern blot analysis of HTLV-1 proviral DNA in the blood is required for the determination of donor. Four factors, including high HTLV-1 proviral loads (PVLs) in the blood, higher age, family history of ATL, and HTLV-1 carriers with any symptoms, were reported to be associated with the risk for ATL development in HTLV-1 carriers (Iwanaga et al., 2010). Donor eligibility of HTLV-1 carriers in allo-HCT for patients with ATL should be determined by considering these risk factors. The recent International Consensus Meeting Report recommends the use of HTLV-1 seronegative donors in allo-HCT for patients with ATL in order to reduce the risk of donor cell-derived ATL after transplantation (Tamaki and Matsuoka, 2006; Cook et al., 2019).

### Preconditioning

Allogeneic hematopoietic cell transplantation for patients with ATL was developed using MAC as for other hematological malignancies. Although the prognosis of patients with aggressive ATL was improved by allo-HCT by using MAC, the TRM rates were very high, ranging from 40 to 56% (Utsunomiya et al., 2001; Kami et al., 2003; Fukushima et al., 2005). Recently, the median age of ATL at diagnosis was reported to be 68 years (Nosaka et al., 2017). Higher age of patients with ATL was probably associated with high TRM rates in patients undergoing allo-HCT by using MAC. In order to reduce the high TRM rates, we conducted prospective clinical trials of non-myeloablative stem cell transplantation (Slavin et al., 1998) for elderly patients with aggressive ATL (≥50 years) by using granulocyte colony-stimulating factor-mobilized peripheral blood stem cells from HLA-matched sibling donors. We succeeded in reducing the TRM rates in about 15% of elderly patients with aggressive ATL who received RIST (Okamura et al., 2005; Tanosaki et al., 2008). Further, we showed that RIST performed using unrelated bone marrow donors was feasible and highly effective for elderly patients with aggressive ATL (Choi et al., 2014). We also showed that cumulative incidence of TRM was 6.7% at 100 days after CBT performed using reduced-intensity conditioning (RIC; Bacigalupo et al., 2009; Giralt et al., 2009; Choi et al., 2016). In the retrospective analysis of the Japanese TRUMP database, the OS rates were not different between patients who were transplanted using MAC, and those who were transplanted using RIC (Ishida et al., 2012a). However, prospective clinical trials considering patient’s age, disease status, and PS at the time of transplantation are needed for the clarification of this issue.

### Outcomes of Allo-HCT

Almost all outcomes of allo-HCT for patients with ATL have been reported by Japanese researchers except one from the European Society for Blood and Marrow Transplantation (EBMT)-Lymphoma Working Party, in which the outcomes of patients receiving transplantation were almost the same as those reported in Japanese patients (Utsunomiya et al., 2001; Hishizawa et al., 2010; Ishida et al., 2012a; Kanda et al., 2012; Bazarbachi et al., 2014; Activities and Outcomes of Hematopoietic Cell Transplantation in Japan, 2017^2^). To our knowledge, in the first report of Japanese nationwide retrospective analysis, we revealed that the 3-year OS rates in patients with ATL receiving allo-HCT from HLA-matched related donors, HLA-mismatched related donors, unrelated donor bone marrow, and unrelated donor cord blood were 41, 24, 39, and 17%, respectively (Hishizawa et al., 2010). Although the prognosis of patients with ATL who underwent CBT was poor previously, the OS rates of patients receiving CBT has gradually improved recently by the decrease of TRM, especially in patients who underwent transplantation in remission (Table 1).

The timing of allo-HCT is very important because obtaining CR is not easy in patients with ATL, and relapse or regrowth of ATL occurs suddenly. The patients who underwent transplantation early (within the first 100 days of diagnosis) showed better outcomes than those who underwent transplantation late (after 100 days of diagnosis). Notably, the survival of patients in non-CR who received early transplantation was significantly better than those who received late transplantation (Fuji et al., 2016a). The rates of ATL-related death were not different in both the groups, but the TRM rates were significantly higher in late transplant patients than in early transplant ones. These results suggest that early transplant is required for better outcomes for patients with ATL in good PS because ATL cells are resistant to anti-cancer agents (Kuwazuru et al., 1990; Ohno et al., 2001a, b); hence, persistent chemotherapy deteriorates patients’ conditions without obtaining CR. When patents with ATL who plan allo-HCT achieve CR or PR, immediate allo-HCT would be recommended after stopping residual courses of chemotherapy.

**FIGURE 3:**
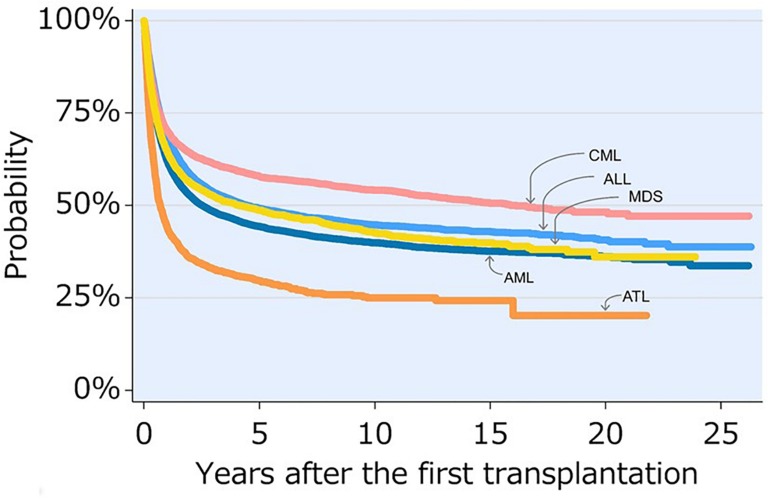
Overall survival after transplant for leukemia 1991–2016. This figure is reproduced from JDCHCT home-page (http://www.jdchct.or.jp/en/data/ slide/2017/). Activities and Outcomes of Hematopoietic Cell Transplantation in Japan (2017) provided by the Japanese Data Center for Hematopoietic Cell Transplantation (JDCHCT). CML, chronic myelogenous leukemia; ALL, acute lymphoblastic leukemia; MDS, myelodysplastic syndrome; AML, acute myelogenous leukemia; ATL, adult T-cell leukemia-lymphoma. Reprinted with permission of the Japanese Data Center for Hematopoietic Cell Transplantation.

Recent Japanese TRUMP data show that the 5-year and 10-year OS rates of patients with ATL who underwent allo-HCT with various kinds of stem cell sources, including bone marrow, peripheral blood stem cell, and cord blood, and conditioning regimens, including MAC and RIC, were 29.6 and 25.0%, respectively (Activities and Outcomes of Hematopoietic Cell Transplantation in Japan, 2017; see text footnote 2). Although the prognosis of ATL patients who underwent allo-HCT is improving, their OS rates are lower than those of patients with other hematological malignancies (Figure 3; Activities and Outcomes of Hematopoietic Cell Transplantation in Japan, 2017; see text footnote 2).

### Prognostic Factors in ATL Patients Who Underwent Allo-HCT

Prognostic factors for poor outcomes such as advanced stage, poor PS, and three continuous variables [age, serum albumin level, and soluble interleukin-2 receptor (sIL-2R) levels] in patients with aggressive ATL were identified using nationwide retrospective analysis, and then the prognostic index (PI) for aggressive ATL (aggressive ATL-PI) was proposed by Katsuya et al. (2012). By using this aggressive ATL-PI, they divided patients into three risk groups (low, intermediate, and high risk; Katsuya et al., 2012). Although aggressive ATL-PI is very useful for the prediction of the prognosis of patients with aggressive ATL, this index was developed by analyzing only patients with aggressive ATL who did not receive allo-HCT. In another nationwide retrospective study that included patients with aggressive ATL aged 70 years or younger who underwent allo-HCT, we identified five prognostic factors for poor outcomes: acute-type ATL, poor PS, high sIL-2R levels (>5,000 U/mL), hypercalcemia (adjusted calcium level ≥12 mg/dL), and high C-reactive protein level (≥2.5 mg/dL). We then developed a new PI: the modified ATL-PI (Fuji et al., 2017). The prognoses of patients were clearly separated according to the three risk groups (low, intermediate, and high-risk). In particular, the OS rates of patients who underwent allo-HCT were significantly higher than those of patients who did not undergo allo-HCT in the intermediate and high-risk groups stratified using the modified ATL-PI (Fuji et al., 2017).

Older age, male sex, non-CR, poor PS, and unrelated HCT were reported to be prognostic factors for poor outcomes in patients with ATL who underwent allo-HCT (Hishizawa et al., 2010). Subsequently, high sIL-2R level at the time of transplantation was also reported to be a poor prognostic factor for disease progression and OS after transplantation (Shigematsu et al., 2014). They also revealed that high sIL-2R level at diagnosis was a poor prognostic factor for OS after transplantation (Shigematsu et al., 2014). We also showed that high sIL-2R was a poor prognostic factor in patients with ATL who underwent allo-HCT by performing a retrospective analysis (Tokunaga et al., 2017). Further, we showed that hematopoietic cell transplantation-specific comorbidity index (HCT-CI; Sorror et al., 2005) and EBMT score (Gratwohl et al., 2009) at transplant were reliable prognostic factors in patients with ATL who underwent allo-HCT (Tokunaga et al., 2017). However, the EBMT score was not associated with the risk of non-relapse mortality (NRM) because about 95% of patients with ATL who underwent allo-HCT were over than 40 years of age in the Japanese TRUMP database (Yoshimitsu et al., 2018a). We attempted to develop another new ATL HCT prognostic index (ATL-HCT-PI). We determined three independent risk factors, including optimized HCT-CI, donor–recipient sex combination (female donor–male recipient), and older age (≥64 years; Yoshimitsu et al., 2018a). By using these three risk factors, we divided patients into 3 risk groups, namely low, intermediate, and high risk, and then developed a new ATL-HCT-PI, which could be used to stratify NRM and OS rates in patients with ATL according to the three risk groups (2-year NRM of 22.0, 27.7, and 44.4%, and 2-year OS of 47.3, 39.1, and 15.7% for ATL-HCT-PI low-, intermediate-, and high-risk patients, respectively; Yoshimitsu et al., 2018a).

As for cytogenetic analysis and clinical significance in patients with ATL, chromosomal abnormalities in patients with ATL were associated with lower OS rates (Itoyama et al., 2001). The OS rates in patients who had multiple chromosomal breaks (at least 6) were significantly poorer than those of patients who had less than 6 chromosomal breaks (*P* < 0.01). The OS rates in patients with abnormal break points in 1p, 1q, 3q, and 17q were significantly poorer than those in patients without these break points (Itoyama et al., 2001). By using the Japanese TRUMP database, we retrospectively analyzed whether chromosomal abnormalities influence the prognosis of patients with ATL, irrespective of whether they underwent allo-HCT, or not. Structural abnormalities such as 2q and 5q were associated with poor prognosis in patients with ATL who underwent allo-HCT (Nakano et al., 2018).

Recently, genetic alterations in ATL cells have been reported in detail (Kataoka et al., 2015). Several genetic alterations are reported to be associated with the prognosis of patients with ATL (Kataoka et al., 2018). In particular, the amplification of PD-L1 and deletion of CDKN2A were detected more frequently in patients with aggressive ATL than in those with indolent ATL, and PKRCB mutation and PD-L1 amplification were poor prognostic factors in patients with aggressive ATL (Kataoka et al., 2018). Unfortunately, the association between genetic alterations and prognosis of patients with ATL who underwent allo-HCT has not been reported. In the future, elucidating whether allo-HCT can overcome these gene alterations associated with poor prognosis in patients with aggressive ATL, or whether patients with these gene alterations can receive allo-HCT is necessary.

## Immune Reaction After Transplantation in ATL

### Graft-Versus-ATL Effect

Graft-versus-leukemia (GVL) reactions were reported to be observed in patients who received allogeneic BMT. GVL reactions are frequently observed in patients complicated with graft-versus-host disease (GVHD); therefore, they are thought to be one of the effects of GVHD. The relapse rates of transplant in identical twins were reported to be higher than those in related siblings without GVHD; hence, GVL reactions were independent phenomena from GVHD (Horowitz et al., 1990). Subsequently, GVHD was reported to induce GVL effect, and the impact on relapse and disease-free survival after transplantation was strong in patients who were transplanted using RIC regimen (Weisdorf et al., 2012).

Immune reactions for tumor cells were occasionally observed in patients with ATL in whom spontaneous regression of ATL occurred (Shimamoto et al., 1993; Matsushita et al., 1999; Takezako et al., 2000). Maintaining remission or another CR after relapse in patients who underwent allo-HCT was thought to be associated with graft-versus-ATL (GV-ATL) effect (Obama et al., 1999; Utsunomiya et al., 2001). We retrospectively analyzed 10 patients with ATL who relapsed after allo-HCT and survived for more than 100 days at our institution. Nine ATL patients relapsed in the skin and other organs, and 5 relapsed only in the skin. Immunosuppressants (ISs) were stopped immediately after the diagnosis of relapse, and then another CR was obtained in 6 patients without chemotherapy and/or donor lymphocyte infusion (DLI). Surprisingly, 4 out of the five patients who had relapsed only in the skin obtained CR only by the cessation of ISs. DLI was applied in two patients who did not show GVHD after the cessation of ISs, and then one patient obtained CR, but died of severe acute GVHD at 5.3 months after transplant. These effects were considered to suggest GV-ATL effects in relapsed patients with ATL after allo-HCT (Yonekura et al., 2008). Itonaga et al. (2013) similarly analyzed the outcomes of 35 patients with relapsed ATL after allo-HCT. They reported that only cytoreductive chemotherapy failed to obtain remission, whereas cytoreductive therapy followed by donor lymphocyte infusion was effective for relapsed ATL in 4 out of 6 patients (Itonaga et al., 2013). This indicates that the reduction of tumor burden requires obtaining GV-ATL effect in patients with ATL who relapsed after allo-HCT.

In patients with ATL who underwent allo-HCT, the prognoses of patients associated with mild acute GVHD (grade I/II) were superior than those of patients without acute GVHD or associated with severe acute GVHD (grade III/IV; Okamura et al., 2005; Kanda et al., 2012; Ishida et al., 2013). As for CBT in patients with ATL, the prognosis of patients associated with mild acute GVHD was slightly better than that of patients without acute GVHD or with severe acute GVHD (Kato et al., 2014). The prognosis of patients with limited chronic GVHD was slightly better than that of patients without chronic GVHD or with extensive chronic GVHD (Kato et al., 2014). These phenomena strongly suggested the existence of GV-ATL effect after allo-HCT in patients with ATL.

### Tax-Specific Cytotoxic T Lymphocyte Response

**FIGURE 4:**
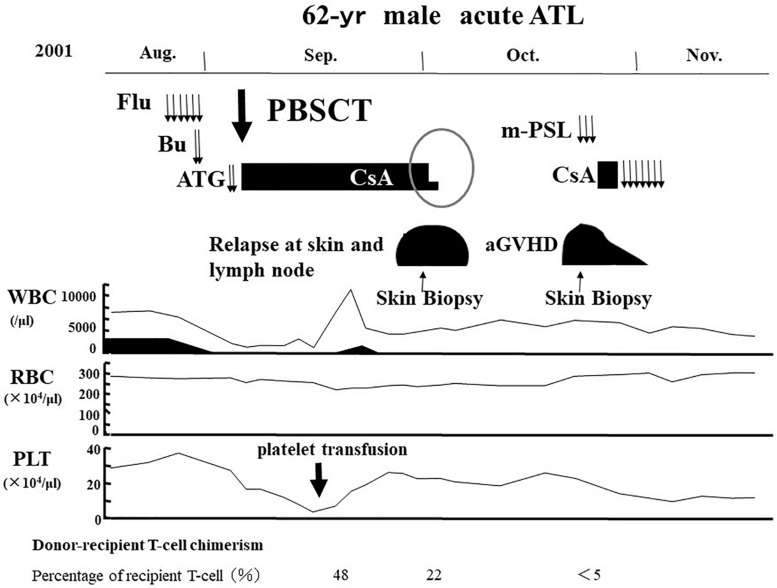
Representative case of graft-versus-ATL effect. ATL, adult T-cell leukemia-lymphoma; Flu, fludarabine; Bu, busulfan; ATG, anti-thymocyte globulin; PBSCT, peripheral blood stem cell transplantation; CsA, cyclosporine A; m-PSL, methylprednisolone; aGVHD, acute graft-versus-host disease; WBC, white blood cell; RBC, red blood cell; PLT, platelet.

**FIGURE 5:**
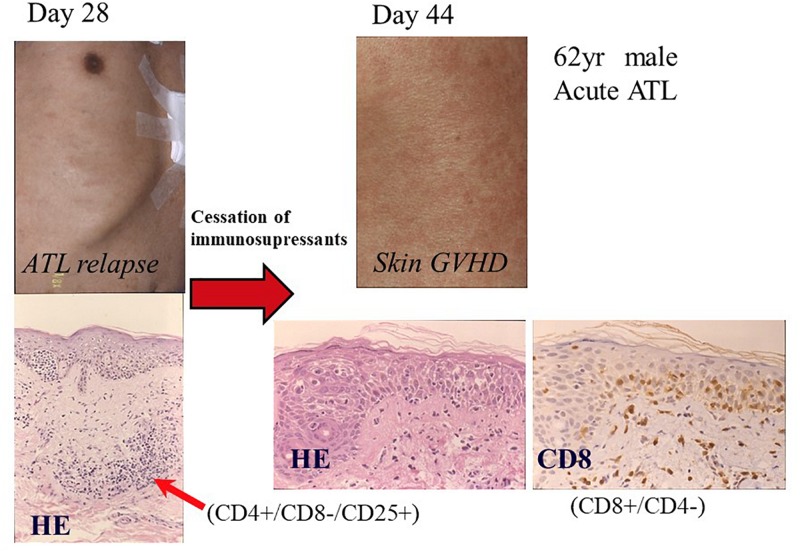
Graft-versus-ATL effect after the cessation of immunosuppressants. Pathological images are reproduced from Figures 3, 4 of Yonekura et al. (2008). Day 28: Skin lesions appeared on day 28 after transplantation (left upper part), which was diagnosed as ATL relapse by skin biopsy. Pathological findings showed ATL cell infiltration in the dermis formed Pautrier’s microabscess and dense dermal infiltration by lymphoid cells with nuclear atypia (left lower portion; HE, hematoxylin-eosin stain; original magnification, ×100). The infiltrates were positive for CD4 and CD25, and negative for CD8. Day 44: Generalized erythema reappeared after disappearance of nodules in the skin after the cessation of immunosuppressants on day 44 after transplantation (right upper portion). Pathological findings by skin biopsy revealed spongiosis, hydropic basal cell degeneration, and dermal edema, which were consistent with acute graft-versus-host disease (HE, original magnification, ×400). Infiltrating lymphocytes were predominantly positive for CD8 (original magnification, ×400). Reprinted by courtesy of Bone Marrow Transplantation.

We reported that another CR was frequently observed in patients with ATL who relapsed after allo-HCT only by the cessation of ISs (Yonekura et al., 2008). One representative case has been shown in Figures 4, 5. A 62-year-old male patient with acute ATL was transplanted from an HLA-matched sibling with anti-HTLV-1 antibody in his serum. He relapsed in the skin, peripheral blood (PB), and lymph nodes 28 days after RIST. IS therapy was stopped immediately after the diagnosis of relapse by skin biopsy. Another CR was observed accompanied by the disappearance of skin lesions, peripheral ATL cells, and superficial lymph nodes. Subsequently, different skin lesions and generalized skin rash appeared in the patient 14 days after the cessation of ISs, and the patient was diagnosed as having Grade II acute GVHD by skin biopsy (Figures 4, 5). Acute skin GVHD was resolved by the administration of steroid hormones and cyclosporine A. Subsequently, CMV infection was detected using the CMV pp65 antigenemia test, and the patient recovered from CMV infection by intravenous ganciclovir administration. The patient was maintained in CR for more than 10 years.

Tax-specific cytotoxic T-lymphocyte (CTL) responses were recognized in the recipient (case mentioned above) PB at the time of another CR after the cessation of ISs; the Tax-specific CTLs were derived from donor lymphocytes (Harashima et al., 2004). Conversely, Tax-specific CTL response was hardly detectable in donor PB (Harashima et al., 2004). In this patient, the Tax-specific CTL responses were maintained for more than 10 years.

Immunotherapy targeting HTLV-1 Tax was considered for long disease control in patients with ATL at least in whom ATL cells expressed Tax, because partial contribution of Tax-specific CTL responses has been suggested to the CR after cessation of ISs in relapsed patients with ATL after RIST (Harashima et al., 2004). Following many basic researches to augment Tax-specific response, Suehiro et al. (2015) conducted a new immunotherapy by using the Tax peptide-pulsed dendritic cell vaccine (Tax-DC vaccine) for patients with aggressive ATL who were controlled in stable condition for at least 1 month by chemotherapy. Three patients were enrolled in that pilot study; by using this immunotherapy, they obtained good clinical responses, which included PR in 2 patients and SD in 1. Responses were maintained for more than 1 year without any additional chemotherapy in the former two patients (Suehiro et al., 2015). At present, phase Ia/Ib study of Tax-DC vaccine has been completed; we intend to start phase II study to investigate the efficacy of this vaccine therapy for patients with ATL.

Interestingly, we also recognized HBZ-specific CD4 T cell responses in patients with ATL after allo-HCT, but not in patients with ATL who did not receive transplant (Narita et al., 2014). In some patients with ATL who underwent allo-HCT, HBZ-specific CD4 T cell responses might have been associated with longer remission after transplant.

### HTLV-1-Infected Cells in the PB After Transplant

Four kinds of HTLV-1 infected cells were found in the PB of patients with ATL who received allo-HCT, i.e., two recipient HTLV-1-infected cells, including residual ATL cells and non-ATL recipient cells, and two donor HTLV-1-infected cells, including cells from HTLV-1 carrier donors and cells infected to donor cells from recipient cells after transplant (Yamasaki et al., 2007; Utsunomiya, 2017). Therefore, HTLV-1-infected cells after allo-HCT do not represent only residual ATL cells but also other HTLV-1-infected cells both from recipient and donor origin. Yamasaki et al. (2007) reported that small number of HTLV-1-infected cells in patients with ATL who underwent allo-HCT were heterogenous and were frequently derived from donors. Considering this fact, we need to evaluate not only disease status but also donor-recipient chimerism; Clonality analysis of HTLV-1 proviral DNA of PB mononuclear cells should be conducted to differentiate ATL relapse from donor-cell derived ATL, when HTLV-1-infected cells increased in the PB after allo-HCT in patients with ATL.

### Kinetics of HTLV-1 Proviral Loads After Transplant

We reported the kinetics of HTLV-1 PVLs in the PB of patients with ATL who received RIST (Choi et al., 2011). In this study, half of donors were HTLV-1 carriers. In patients who were transplanted from HTLV-1 carrier donors, HTLV-1 PVLs in the PB were maintained in relatively high levels consistently because HTLV-1-infected cells were transplanted to recipients together with hematopoietic stem cells. In patients who were transplanted from HTLV-1 negative normal donors, HTLV-1 PVLs markedly decreased in most cases, and two types of HTLV-1 PVL fluctuation patterns were noted: first, HTLV-1 PVLs in PB became detectable, which were equivalent to HTLV-1 carrier levels; second, HTLV-1 PVLs were maintained in undetectable levels for a long time (Choi et al., 2011).

Although HTLV-1 PVLs in the PB do not represent only residual ATL cells after transplant, evaluation of the kinetics of HTLV-1 PVLs in ATL patients who underwent allo-HCT is useful for detecting the early ATL relapse and diagnosis of donor cell-derived ATL in combination with donor-recipient chimerism analysis of peripheral lymphocytes.

The eradication of HTLV-1 by allo-HCT has been reported in a young male patient with congenital pure red-cell anemia (Kawa et al., 1998). HTLV-1 PVLs frequently disappeared from the PB after allo-HCT in patients who were transplanted from HTLV-1 negative donors, but thereafter small number of HTLV-1-infected cells frequently appeared in the PB (Yamasaki et al., 2007; Choi et al., 2011). Prevention of HTLV-1 infection to donor cells from recipients who received transplant from HTLV-1 negative donors should be considered by administering antiviral drugs after transplant.

## Complications of Transplant for ATL

### Early Complications After Allo-HCT in ATL: Focusing on Viral Infection

Adult T-cell leukemia-lymphoma patients are severely immunocompromised and have frequent complications of opportunistic infections, including various kinds of viral infection. In patients with ATL, CMV, Epstein–Barr virus, and human herpes virus-6 (HHV-6) infections were frequently observed after cytotoxic chemotherapy (Ogata et al., 2011). High incidence of CMV infection was reported in patients with ATL after allo-HCT (Nakano et al., 2014). CMV infection has been reported to be associated with poor prognosis of patients with ATL who received allo-HCT (Sawayama et al., 2019). HHV-6 infection has also been reported in patients with ATL after CBT or unrelated BMT (Ogata et al., 2008, 2013). Monitoring for CMV antigenemia and HHV-6 in ATL patients who undergo allo-HCT is necessary for the early detection and empirical therapy for these viral infections. There is no report that empirical therapy for HHV-6 can reduce the incidence of HHV-6 infection and TRM in patients with ATL who underwent allo-HCT. However, patients who developed HHV-6 encephalitis may have aggravation of symptoms within a short period (Ogata et al., 2013). There is a report that prophylaxis for HHV-6 using foscarnet reduced the incidence of HHV-6 infection where it is possible that HHV-6 prophylaxis prevented the progression of HHV-6 encephalitis; nevertheless, the incidence of encephalitis did not decrease (Ogata et al., 2018). Empirical therapy for HHV-6 infection in patients with ATL who received allo-HCT is recommended.

### Late Complications: Focusing on Donor Cell Leukemia and Lymphoma

Donor cell leukemia (DCL) has been reported in patients who underwent allo-HCT for various kinds of hematological malignancies (Wiseman, 2011). Although DCL was thought to be a very rare complication previously, about 5% of patients who underwent allo-HCT were recently reported to develop DCL (Wiseman, 2011). One of the reasons for the increase of DCL is thought to be the increase of donor age because of the relevance of RIC regimen for allo-HCT. The risk of DCL might be high in patients who undergo allo-HCT because of the long survival time.

Two cases of donor cell-derived ATL have been reported (Tamaki and Matsuoka, 2006; Nakamizo et al., 2011). Both patients were transplanted from HTLV-1 carrier donors. One patient developed donor cell-derived ATL 133 days after transplant. DCL was thought to be derived from HTLV-1-infected donor cells because ATL occurred in considerably short time after allo-HCT. In this case, HTLV-1-infected abnormal clone was not detected by Southern blot analysis of PB lymphocytes in the donor (Tamaki and Matsuoka, 2006). The occurrence of donor cell-derived ATL after allo-HCT, especially in the case of transplantation from HTLV-1 carrier donor, should be paid special attention.

Two cases of DCL/lymphoma other than ATL were reported: one was a patient with acute myelogenous leukemia, and the other was Burkitt lymphoma that occurred about 11 years after allo-HCT (Matsunaga et al., 2005; Takeuchi et al., 2015).

## New Strategy for ATL by Using Transplant

### New Agents for Allogeneic Hematopoietic Cell Transplantation in ATL

Recently, new molecular targeting agents such as anti-CC chemokine receptor 4 (CCR4) antibody, mogamulizumab, and lenalidomide have been approved for ATL patients in Japan.

First, we showed that approximately 90% of patients with ATL had CCR4-positive ATL cells, and the survival of CCR4-positive patients was lower than that of patients without CCR4 expression (Ishida et al., 2003, 2004b). Defucosylated chimeric anti-CCR4 monoclonal antibody, KM2760, which enhanced antibody-dependent cellular cytotoxicity (ADCC), was developed in Japan (Niwa et al., 2004). We showed that KM2760 had strong cytotoxicity for fresh ATL cells derived from patients with ATL in the autologous setting by using patients’ effector cells (Ishida et al., 2004a). Subsequently, humanized anti-CCR4 monoclonal antibody, KW-0761/mogamulizumab was further developed in Japan; we showed that mogamulizumab had the same strong cytotoxic effect for fresh ATL cells derived from ATL patients in the same autologous setting as that for KM2760 (Ishii et al., 2010). Based on several preclinical experiments of mogamulizumab for ATL cells, we conducted two prospective clinical studies (phases I and II) for patients with relapsed aggressive ATL in order to investigate the safety and efficacy of mogamulizumab. These two prospective studies revealed acceptable safety and good efficacy of mogamulizumab for patients with relapsed aggressive ATL. Mogamulizumab was thus approved for patients with relapsed or refractory ATL in Japan (Yamamoto et al., 2010; Ishida et al., 2012b). We further conducted a randomized study to evaluate the efficacy of VCAP–AMP–VECP [vincristine, cyclophosphamide, doxorubicin, and prednisone (VCAP)-doxorubicin, ranimustine and prednisone (AMP)–vindesine, etoposide, carboplatin and prednisone (VECP)] therapy alone (Tsukasaki et al., 2007) versus that in combination with mogamulizumab for newly diagnosed patients with aggressive ATL. We found that the CR rate in combination therapy was higher than that in VCAP–AMP–VECP therapy alone (52 vs. 33%; Ishida et al., 2015). Thus, mogamulizumab was approved for patients with newly diagnosed aggressive ATL in combination with chemotherapy in Japan in 2014.

The higher CR rates of patients with ATL who were treated with mogamulizumab in combination with chemotherapy were expected to improve the prognosis of patients who received allo-HCT. Unexpectedly, nationwide retrospective analysis revealed that mogamulizumab administration before allo-HCT for patients with ATL was associated with an increased risk of severe, acute, steroid-resistant GVHD, as well as higher NRM at 1 year. The 1-year OS in patients who had used mogamulizumab before allo-HCT was significantly lower than that in patients treated without mogamulizumab (Fuji et al., 2016b). When the OS and TRM rates were analyzed according to the interval between the last mogamulizumab administration and allo-HCT, the TRM rates decreased and the OS rates improved in patients in whom the interval was equal to and more than 50 days and were equivalent to those in patients not receiving mogamulizumab (Fuji et al., 2016b). Sugio et al. (2016) also retrospectively analyzed 25 patients with ATL receiving mogamulizumab before allo-HCT. Severe acute GVHD (Grade III/IV) was observed in 34.7% patients. By analyzing the interval between the last administration of mogamulizumab and allo-HCT, they showed that the frequency of severe acute GVHD (Grade III/IV) was significantly higher in patients receiving mogamulizumab within 80 days before transplant than in those having more than 80-day interval (Sugio et al., 2016).

Although a prospective randomized study revealed that the CR rate of mogamulizumab in combination with VCAP-AMP-VECP therapy was significantly higher than that of VCAP-AMP-VECP therapy alone, the OS in both groups was not significantly different (Ishida et al., 2015). Of the 53 patients enrolled in that clinical trial, 12 underwent allo-HCT after protocol therapy. Subsequent follow-up study revealed that the OS in patients who underwent transplant who did not receive mogamulizumab before allo-HCT was better than that of patients who received mogamulizumab before transplant (Ishida et al., 2019). Mogamulizumab can decrease not only CCR4-positive ATL cells but also normal regulatory T cells expressing CCR4. The half-life of plasma concentration after the eighth administration of mogamulizumab at 1.0 mg/kg is reported to be about 18 days, suggesting that several months are required for the disappearance of mogamulizumab in PB after intravenous administration (Ishida et al., 2012b). Mogamulizumab induced strong cytotoxic effect for CCR4-positive cells in low serum concentration because the ADCC activity of mogamulizumab was markedly enhanced by the defucosylation of IgG Fc portion of antibodies (Ishida et al., 2004a; Niwa et al., 2004). The decrease of normal regulatory T-cells might be associated with an increased risk of severe acute GVHD after transplant and poor prognosis of patients with ATL who underwent allo-HCT.

Conversely, at our institution, the 1-year OS rate of nine patients with ATL who underwent allo-HCT after 1–2 times of mogamulizumab administration was not less than that of 113 patients who did not receive mogamulizumab (55.6 vs. 37.2%, *P* = 0.843; Fuji et al., 2018a). Recently, hematologists and transplant physicians in Japan discussed the appropriate use of mogamulizumab for patients with aggressive ATL who planned to undergo allo-HCT, regarding the frequency and timing of mogamulizumab administration and the interval between the last mogamulizumab administration and transplantation. Thus, we proposed the appropriate use of mogamulizumab for patients with aggressive ATL before allo-HCT as follows: (1) mogamulizumab should not be administered to patients with aggressive ATL who plan to undergo transplantation if patients can obtain CR or PR without mogamulizumab; (2) mogamulizumab should be administered a few times only when patients cannot obtain remission by ordinary combination chemotherapy; (3) allo-HCT should be recommended with at least 50-day interval, if possible, between last mogamulizumab administration and transplant, and (4) no consensus about GVHD prophylaxis, choice of stem cell source and prophylaxis for infectious diseases (Fuji et al., 2018a).

Anti-CC chemokine receptor 4 mutations were detected in 14/53 ATL samples (26%) and consisted exclusively of non-sense or frameshift mutations that truncated the coding region at C329, Q330, or Y331 in the carboxy terminus (Nakagawa et al., 2014). They showed that the CCR4-Q330 non-sense isoform was a gain-of-function mutation. Another group analyzed CCR4 gene alterations in patients with ATL, and gene mutations were found in 27% of ATL patients who had frameshift and non-sense mutations. The frameshift mutations in patients with ATL were associated with poor prognosis (Yoshida et al., 2016). We also analyzed CCR4 gene alterations in 116 patients with ATL; CCR4 mutations were found in 33% of the patients. Mogamulizumab therapy was extremely effective for ATL patients with CCR4 mutations. CCR4 mutation status had no significant impact in patients who neither receive mogamulizumab-containing treatment nor allo-HCT. OS in patients with CCR4 mutations who did receive mogamulizumab-containing treatment but no allo-HCT was significantly superior to that in patients without CCR4 mutations (Sakamoto et al., 2018). These results suggest that mogamulizumab therapy can obtain long-term survival in ATL patients with CCR4 mutations. In the near future, CCR4 mutations in patients with ATL need to be detected because they might influence the decision of therapeutic strategies for patients with ATL, including transplantation.

Another new drug, lenalidomide is useful for patients with ATL. Lenalidomide is one of the key drugs for multiple myeloma (MM) therapy. Clinical trials of lenalidomide in phases I and II revealed the efficacy for patients with relapsed ATL, and then lenalidomide was approved for patients with relapsed ATL in Japan in 2017 (Ishida et al., 2016; Ogura et al., 2016). Unfortunately, the efficacy of lenalidomide for patients with ATL who underwent transplant has not been reported in detail, although it is expected to be one of the promising key drugs for salvage therapy for relapsed patients with ATL. In MM patients, lenalidomide for maintenance therapy to prevent relapse or progression after non-myeloablative allo-HCT was not feasible because of the increased acute GVHD (Kneppers et al., 2011). Conversely, another study showed that small amount of lenalidomide was feasible for MM patients after allo-HCT, although the frequency of acute GVHD was high (Alsina et al., 2014). Further, a study showed that half of the MM patients who relapsed after allo-HCT could be controlled by lenalidomide administration (Bensinger et al., 2014). Only one patient with ATL was reported to have been successfully treated by a small dosage of lenalidomide for relapse after allo-HCT (Ando et al., 2018). The appropriate use of lenalidomide could be useful for patients with ATL who relapsed after allo-HCT or who planned to undergo allo-HCT. The therapeutic strategy for using lenalidomide for patients with ATL who received allo-HCT should be established in the near future.

### New Methods of Allo-HCT for ATL

Haploidentical HCT was performed for highly aggressive hematological malignancies by using non- MAC because of the decrease in HLA-matched related siblings and long period for coordination in unrelated HCT cases (Ogawa et al., 2006). Recently, an approach using post-cyclophosphamide administration in haploidentical HCT has become popular (Luznik et al., 2008; Kasamon et al., 2010; Sugita et al., 2015).

Recently, we evaluated the prognosis of haploidentical HCT for ATL patients without post-cyclophosphamide administration by using Japanese TRUMP database. Although the TRM rate of haploidentical HCT without post-cyclophosphamide administration was very high, which included 1-year non-ATL-related death of 41.3%, about 20% for the patients survived for more than 5 years (Yoshimitsu et al., 2018b). The safety and efficacy of haploidentical HCT was investigated by two prospective multicenter clinical studies in Japan that are underway and are using both MAC and RIC for patients with ATL followed by post-cyclophosphamide administration. The outcomes of haploidentical HCT for patients with ATL without post-cyclophosphamide administration reported by Yoshimitsu et al. (2018b) are very important compared to those of patients who underwent haploidentical HCT followed by post-cyclophosphamide administration. Because ATL known to occur at higher ages (median age, about 68 years; Nosaka et al., 2017), developing haploidentical HCT by using RIC for patients with ATL followed by post-cyclophosphamide administration is necessary.

## Future Direction

Allogeneic hematopoietic cell transplantation is an immunotherapy based on allogeneic immune reactions after transplant. Conversely, autologous stem cell transplantation is an intensive chemotherapy that involves numerous anti-cancer agents and/or radiotherapy. Therefore, the time to obtain immunological effects against tumor cells and reduction of tumor volume before allo-HCT are necessary for obtaining remission. In particular, ATL is an aggressive hematological malignancy that can relapse immediately even in CR. Immunological effects in patients with aggressive ATL can be obtained by determining the disease status and PS at the time of HCT. Strong immune responses after transplant can be induced using allo-HCT in combination with pure immunotherapy without stem cell transplant to produce good prognoses in patients with ATL in the near future. Furthermore, new strategies of allo-HCT for patients with ATL are is needed for the improving the cure rates of patients.

## Conclusion

The history and progression of allo-HCT for patients with ATL are reviewed. Although allo-HCT procedures for patients with ATL have markedly progressed, which include the development of RIC, haploidentical transplant, and diversity of indications for allo-HCT, the TRM rates after transplant remain considerably high owing to older age of patients with ATL and high frequency of infectious complications. Further development of allo-HCT by using new molecular targeting agents is required for the improvement of the cure rates of patients with ATL.

## Author Contributions

AU designed the study and wrote the draft of the manuscript.

## Conflict of Interest

AU received personal fees from the Kyowa Hakko Kirin, and Celgene, outside the submitted work.

## Footnotes

1 http://www.jdchct.or.jp/data/report/2017/

2 http://www.jdchct.or.jp/en/data/slide/2017/
